# Pituitary Adenylate Cyclase-Activating Polypeptide Protects Glomerular Podocytes from Inflammatory Injuries

**DOI:** 10.1155/2015/727152

**Published:** 2015-03-02

**Authors:** Kenichi Sakamoto, Kyoko Kuno, Minoru Takemoto, Peng He, Takahiro Ishikawa, Shunichiro Onishi, Ryoichi Ishibashi, Emiko Okabe, Mayumi Shoji, Akiko Hattori, Masaya Yamaga, Kazuki Kobayashi, Harukiyo Kawamura, Hirotake Tokuyama, Yoshiro Maezawa, Koutaro Yokote

**Affiliations:** ^1^Department of Clinical Cell Biology and Medicine, Chiba University Graduate School of Medicine, Japan; ^2^Division of Diabetes, Metabolism and Endocrinology, Chiba University Hospital, Japan

## Abstract

Diabetic nephropathy (DN) is a leading cause of end-stage kidney disease; however, there are few treatment options. Inflammation plays a crucial role in the initiation and/or progression of DN. Pituitary adenylate cyclase-activating polypeptide (PACAP) is a neuropeptide, which was originally isolated from the ovine hypothalamus and reportedly has diverse biological functions. It has been reported that PACAP has renoprotective effects in different models of kidney pathology. However, the specific cell types within the kidney that are protected by PACAP have not yet been reported. In this study, we localized VPAC1, one of the PACAP receptors, to glomerular podocytes, which also reportedly has crucial roles not only in glomerular physiology but also in pathology. PACAP was effective in the downregulation of proinflammatory cytokines, such as monocyte chemoattractant protein-1 (MCP-1) and interleukin-6, which had been induced by the activation of toll-like receptor (TLR) with lipopolysaccharide. PACAP also had downregulated the expression of MCP-1 through the protein kinase A signaling pathway; this led to the attenuation of the activation of extracellular signal-regulated kinase and nuclear factor-kappa B signaling. Our results suggested that PACAP could be a possible treatment option for DN through the use of anti-inflammation effects on glomerular podocytes.

## 1. Introduction

The prevalence of diabetes has explosively increased worldwide, leading to an increase in the number of patients who suffer from diabetic vascular complications such as diabetic nephropathy (DN) [1]. DN is not only the leading cause of end-stage kidney disease but also a significant risk factor for cardiovascular disease [2]. Although the treatment for DN is important to improve patients' prognosis, the current treatment remains suboptimal and therefore novel approaches for DN are urgently needed.

Dipeptidyl peptidase-4 inhibitors (DPP4i) have been recently introduced in clinic as a new oral hypoglycemic agent. DPP4 is a serine exopeptidase and processes the substrates that have either N-terminal proline or alanine including glucagon-like peptide-1 (GLP-1) and glucose-dependent insulinotropic polypeptide (GIP) known as incretin hormones. These incretin hormones are secreted from the small intestine after foods intake and induce the release of insulin from the pancreatic beta cells in the islets of Langerhans. In addition to the reduction in the blood glucose level, it has been reported that DPP4i possesses properties and can protect the cardiovascular system [3], kidney [4], liver [5], and bone [6] from injuries. Since it has been reported that receptors for incretins were not expressed within glomerulus [7], protective effects of DPP4i for kidney might depend on another substrate for DPP4. For instance, stromal cell-derived factor-1 (SDF-1), one of the substrates for DPP4, can be stabilized by DPP4i [8]. Because SDF-1 is the most important protein for the recruitment and homing of bone marrow-derived regenerative stem cells, increased levels of SDF-1 through DPP4 inhibition has reportedly increased the intragraft number of progenitor cells that had contributed to the recovery from ischemia-reperfusion lung injury throughout the mammalian system [8]. It has also been reported that DPP4i decreased the levels of urinary albumin excretion in diabetic patients [9, 10]; however, the mechanism behind how DPP4i ameliorates kidney injuries is not yet clear.

Pituitary adenylate cyclase-activating polypeptide (PACAP) is one of the substrates of DPP4. PACAP belongs to the glucagon superfamily of peptides and was originally purified from sheep hypothalamus in 1989 [11]. PACAP is able to potentiate cyclic adenosine monophosphate (cAMP) production in pituitary cells and has a diverse array of biological functions, particularly neuroprotective and general cytoprotective roles, such as anti-apoptosis and anti-inflammation [12]. Although the highest concentrations are observed in the nervous system, a wide variety of tissues, such as heart, pancreas, liver, and kidney, produce PACAP; secreted PACAP also has protective effects on the different types of tissues and cells through the three different receptors: PAC1, VPAC1, and VPAC2. PACAP was found to exist in two forms: 38-amino-acid, a major form, and 27-amino-acid, a short one, truncated at C-terminal and to a much lesser extent in blood stream, respectively. Recently, it has been reported that DPP4 degraded PACAP (1–27) and (1–38) to form PACAP (3–27) and (3–38) [13]. These DPP4-produced metabolites of PACAP reportedly lost their biological activity. It has been reported that PACAP has protective effects in the kidney against various insults, including ischemia/reperfusion injury [14], drug-induced nephrotoxicity [15], and myeloma light chain-induced nephropathy [16]. PACAP-deficient mice were highly susceptible to renal ischemia/reperfusion [17]. PACAP treatment in streptozotocin-induced diabetic animals decreased cytokine expression and prevented kidney injuries [18]. These results indicated that PACAP has protective roles in the kidney; however, it is unknown which cell types PACAP affects and how it decreases cytokine expression. Therefore, in this study, we investigated the effects of PACAP on kidney cells and the mechanisms of how PACAP decreases the expression of inflammatory cytokines.

## 2. Materials and Methods

### 2.1. Materials

Human recombinant PACAP1-38 was obtained from Tocris bioscience. Lipopolysaccharide (LPS) and U-73122 (phospholipase C (PLC) inhibitor) were obtained from Sigma Aldrich and H89 (protein kinase A (PKA) inhibitor) was obtained from Cell Signaling.

### 2.2. Cell Culture

Cultured mouse podocytes, transformed by ectopic expression of cyclin-dependent kinase 4, were kindly provided by Dr. Toru Sakairi (Gunma University Graduate School of Medicine, Japan) [19]. Podocytes were maintained in Roswell Park Memorial Institute (RPMI) 1640 medium containing 10% fetal bovine serum (FBS, Sigma Aldrich, USA), penicillin (100 U/mL), and streptomycin (100 U/mL, Sigma Aldrich, USA). The podocytes were cultured at 37°C in humidified air containing 5% CO_2_.

### 2.3. Animals

All procedures were performed in accordance with the guidelines of the Research Center for Animal Life Science of Chiba University of Medical Science. Six- to nine-week-old male C57Bl/6J mice were purchased from CLEA Japan (Japan) and housed in cages and maintained on a 12 h light/12 h dark cycle.

### 2.4. Isolation of Glomeruli

The glomeruli were isolated by Dynabeads (invitrogen, Norway) perfusion technique as previously described [20].

### 2.5. Immunohistological Assessment

The kidney tissues were dissected from the mouse, fixed in OCT compound, and stored at −80°C until use. Several 6 *μ*m thick frozen sections were prepared and fixed with ice-cold methanol, air-dried for 30 min at room temperature, and then blocked with blocking buffer containing 2% bovine serum albumin (BSA) and 0.05% Tween-20 in PBS. After washing with Tween in PBS (PBST; 0.1% Tween-20 in PBS), the slides were coincubated with VPAC1 antibody (1 : 50 dilution, Santa Cruz: sc-30019), podocalyxin (1 : 200 dilution, R&D systems: MAB1556), and toll-like receptor 4 (1 : 50 dilution, Santa Cruz: sc-10741). The slides were imaged by the Axio Observer D1 (ZEISS) or with a confocal laser scanning microscope (Leica LSM5 PASCAL). The images were processed using Adobe Photoshop.

### 2.6. Immunocytochemistry

The podocytes were cultured in Lab-Tek II chamber slides (Nalge Nunc International), deprived of serum for 24 h, and stimulated with 100 ng/mL LPS for 1 h. Then the podocytes were fixed in ice-cold methanol for 10 min, rinsed with PBS, and incubated in a blocking buffer containing 0.5% BSA and 0.25% Tween-20 in PBS for 1 h at room temperature. The slides were then incubated with an anti-nuclear factor-kappa B (NF-*κ*B) antibody (1 : 50, Santa Cruz: sc-372) overnight at 4°C, washed several times with PBS, and then incubated with 1 : 1000 dilution of fluorescent-conjugated secondary antibody (Alexa Fluor 488 goat anti-rabbit IgG, Invitrogen) for 1 h at room temperature. The slides were then washed with PBST, nuclear-stained with Hoechst 33342, and mounted with a fluorescence mounting medium. For the evaluation of immunostaining for NF-*κ*B, the cells that had accumulated NF-*κ*B in their nuclei were counted at 10 randomly selected areas. The results were expressed as fold changes relative to controls.

### 2.7. RT-PCR and Quantitative PCR

The total RNA was extracted using the PureLink RNA Mini kit (Ambion: 12183-018A) according to the manufacturer's protocols. Two micrograms of total RNA were reverse-transcribed with the SuperScript III reverse transcriptase kit (Invitrogen: 18080). The complementary DNA product was then subjected to PCR using the system with different pairs of oligonucleotide primers that were shown in Supplemental Table 1, available online at http://dx.doi.org/10.1155/2015/727152. The cycling conditions were as follows: a denaturation step at 94°C for 30 s followed by 30 cycles, annealing at 55°C for both PAC1 and *β*-actin and 58°C for VPAC1 for 30 s, and elongation at 72°C for 30 s with the final extension at 72°C for 7 min. The PCR products were separated by gel electrophoresis on a 2% agarose gel with ethidium bromide; the signals were quantified using a ChemiDoc MP ImageLab PCsystem (BIO-RAD).

Quantitative PCR was performed in the 7500 Fast Real-Time PCR system (Applied Biosystems) using the Fast SYBR Green Master Mix (Applied Biosystems: 4385612). The primers used were shown in Supplemental Table 1. PCR conditions were set for incubation at 95°C for 20 s followed by 40 cycles of 3 s at 95°C and 30 s at 60°C.

### 2.8. Immunoblotting

Podocytes were lysed in a SDS sample buffer containing 0.5 M Tris-HCl, 10% SDS, glycerol, bromophenol blue, and 3% 2-mercaptoethanol. They were boiled at 95°C for 10 min, and then the protein was fractionated on 10%–15% polyacrylamide gels (e-PAGEL, ATTO Corporation, Japan). The protein was transferred to PVDF membranes (Immobilon-P Transfer Membrane); the membranes were blocked for 1 h at room temperature to block the nonspecific binding of the protein and incubated for 18 h at 4°C with primary antibodies. The primary antibodies that were used were as follows: anti-VPAC1 antibody (1 : 200 dilution, Santa Cruz: sc-30019), anti-phospho-p44/42 MAPK (extracellular signal-regulated kinase (ERK1/2)) antibody (1 : 1000 dilution, Cell-Signaling: number 9106), and anti-p44/42 MAPK (ERK1/2) antibody (1 : 1000 dilution, Cell-Signaling: number 9102). The blots were then washed and incubated with second antibodies, peroxidase-conjugated anti-rabbit immunoglobulins (1 : 2500 dilution, GE healthcare), or goat anti-mouse IgG-HRP (1 : 2500 dilution, Santa Cruz: sc-2055) for 1 h at room temperature. After washing for several times, the antibody binding sites were visualized using an ECL western blotting detection system (GE healthcare: RPN2106). The blots were quantified using a ChemiDoc MP ImageLab PCsystem (BIO-RAD).

### 2.9. Luciferase Assay

Podocytes were plated in 96-well dishes at 50% confluency and transfected with 10 ng per well cAMP response element- (CRE-) lux construct that contained consecutive cAMP response element by Lipofectamine LTX and PLUS Reagents (Invitrogen). At Six hours after transfection, cells were deprived of serum for 48 h. And then, the cells were stimulated with PACAP for 1 h. The luciferase activities in the cell lysate were measured in a 1420 ARVO SX multilabel counter (Wallac, Inc., Gaithersburg, MD) using the Dual Luciferase Reporter Assay System (Promega, Madison, WI). To correct for potential variation in transfection efficiency, pRL-TK vector (Promega) was cotransfected in all experiments. All assays were performed in quadruplicate and repeated six times.

### 2.10. Statistical Analyses

Statistical analyses were done using SAS 9.3 and/or Microsoft Office Excel by Student's unpaired *t*-test. All experiments were repeated at least three times. The data were presented as the mean ± SEM. *P* values < 0.05 were considered statistically significant.

## 3. Results

### 3.1. A PACAP Receptor, VPAC1, Was Expressed in Glomerular Podocytes

**Figure 1:**
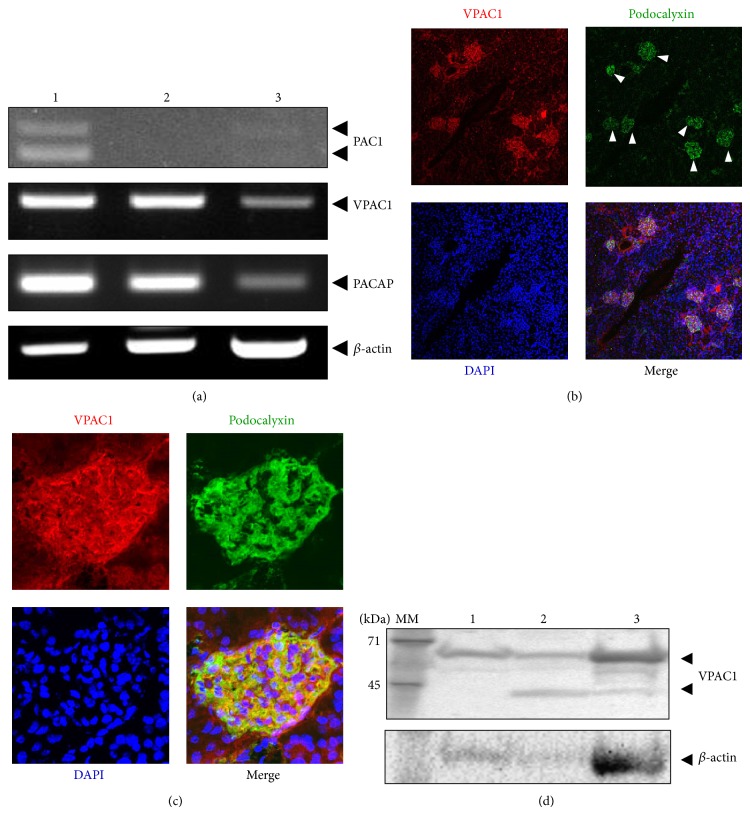
The expression of PACAP receptors within kidney. (a) The expression of PACAP/VIP family receptors transcripts were evaluated by RT-PCR. Lane 1: RNA from cerebellum used for positive control; lane 2: RNA from isolated glomeruli; lane 3: RNA from cultured podocyte. ((b), (c)) Immunohistochemistry using anti-VPAC1 antibody showed that VPAC1 protein was localized in glomeruli ((b), low magnification) and primarily localized in podocytes ((c), high magnification). Anti-podocalyxin antibody was used as a marker for podocytes. Arrowheads indicated glomeruli. (d) Expression of VPAC1 protein was evaluated by immunoblotting using anti-VPAC1 specific antibody.

PACAP works as a ligand and binds with specific receptors in order to transduce intracellular signals. Therefore, we first examined in which cell types PACAP transduces intracellular signal within the glomeruli. Reverse transcriptase PCR revealed that mRNA for VPAC1 but not PAC1 was detected in glomeruli as shown in Figure 1(a). Immunohistochemistry revealed that VPAC1 was primarily expressed in podocalyxin-positive cells (Figures 1(b) and 1(c)). Complete absence of signal was observed when primary antibodies were omitted (data not shown). RT-PCR (Figure 1(a)) and western blotting (Figure 1(d)) also confirmed that the VPAC1 mRNA and protein were expressed not only in isolated glomeruli but also in cultured podocytes. We were not able to detect VPAC2 mRNA probably due to technical reasons.

### 3.2. PACAP Increased the cAMP and Activated cAMP Downstream Target

**Figure 2:**
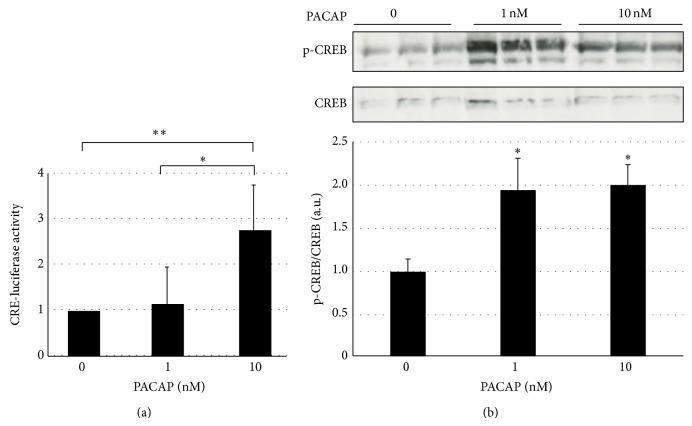
PACAP increased the promoter activities of cAMP responsive element and activated cAMP responsive element binding protein. (a) The promoter activities of cAMP responsive element were measured within podocytes in the presence or absence of PACAP. ^*^ *P* < 0.05; ^**^ *P* < 0.01; experiments were repeated at least six times and bars represented mean ± SD. (b) Podocytes were treated with different concentrations of PACAP as indicated in figure for 1 h. Then cells were lysed and subjected to western blot analysis for phospho-CREB (normalized in all cases to total CREB). ^*^ *P* < 0.05 versus control. Experiments were repeated at least three times and bars represented mean ± SD.

Because VPAC1, a PACAP receptor, was expressed on podocytes, we next examined whether PACAP acted on podocytes. It has been reported that PACAP binds with VPAC1, which is coupled with Gs protein and induces rapid cAMP production, which ultimately activates the PKA pathway. Ten nM PACAP significantly increased the cellular contents of cAMP in a time dependent manner (Supplemental Figure 1). Increased levels of cAMP in the presence of PACAP was associated with increased levels of cAMP responsive element promoter activities (Figure 2(a)) and phosphorylated cAMP response element binding protein (CREB) (Figure 2(b)). These results indicated that PACAP primarily sent signals to glomerular podocytes, especially activating the cAMP/PKA pathway in the podocytes.

### 3.3. PACAP Attenuated the Expression of the Proinflammatory Cytokines Induced by Lipopolysaccharide (LPS) through PKA Signaling

**Figure 3:**
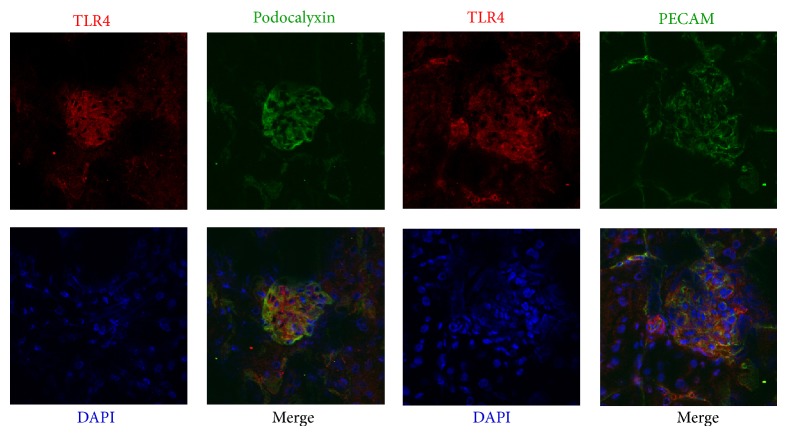
The expression of toll-like receptor 4 (TLR4) in murine kidney. Immunohistochemistry using anti-TLR4 antibody showed that TLR4 protein was localized to both podocytes and endothelial cells. Anti-podocalyxin antibody was used as a marker for podocytes and anti-PECAM antibody was used as a marker for endothelial cells.

**Figure 4:**
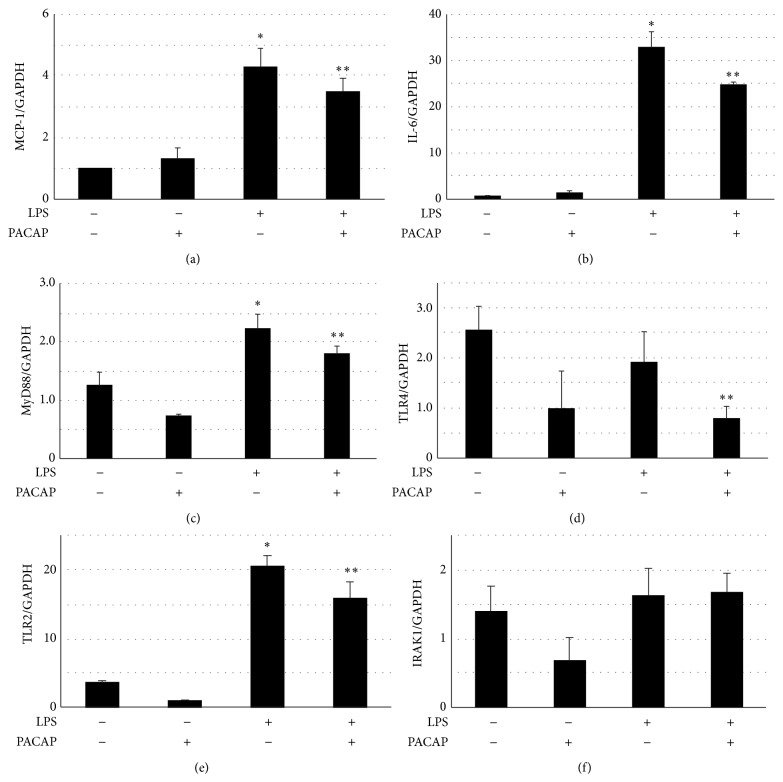
Effects of PACAP on the expression of inflammatory cytokines and TLR-related genes in podocytes. Podocytes were pretreated with 10 nM PACAP for 1 h and then stimulated with 0.1 *μ*g/mL LPS for 3 h. The expressions of MCP-1, IL-6, TLR4, TLR2, MyD88, and IRAK1 mRNA were analyzed by real-time PCR. GAPDH was used as an internal standard. ^*^ *P* < 0.001 compared with the control group. ^**^ *P* < 0.05 compared with the LPS group. Experiments were repeated at least three times and bars represented mean ± SD.

**Figure 5:**
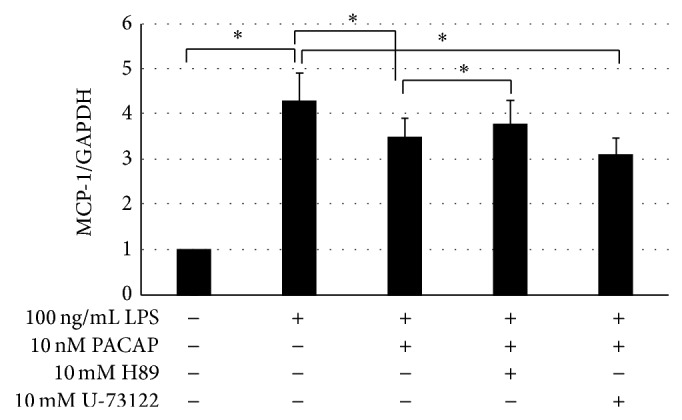
Effect of PKA inhibitor and PLC inhibitor on the expression of MCP-1 induced by LPS. Podocytes were pretreated with PACAP with or without 10 mM H89 (PKA inhibitor) or U-73122 (PLC inhibitor) for 1 h and then stimulated with 0.1 *μ*g/mL LPS for 3 h. The expression of MCP-1 mRNA was analyzed by RT-PCR. GAPDH was used as internal standard.  ^*^indicated *P* < 0.05. Experiments were repeated at least three times and bars represented mean ± SD.

It has been reported that PACAP also played an anti-inflammatory role in peripheral and central tissues. We examined the effects of PACAP on the proinflammatory signaling in podocytes. Toll-like receptors (TLRs) are the principal mediators of innate immunity and are reportedly activated by bacterial endotoxins. The TLR4 proteins were localized to podocytes and endothelial cells by immunohistochemistry as shown in Figure 3. Then, we examined the effects of PACAP on the expression of inflammatory cytokines. As shown in Figure 4, LPS, a ligand of TLR4, significantly increased the expression of IL-6 and MCP-1. In the presence of PACAP, the increased expressions of IL-6 and MCP-1 were significantly attenuated. The expression of TLR2, TLR4, and myeloid differentiation primary response gene 88 (MyD88), a TLR adaptor protein, but not that of IL-1 receptor associated kinase-1 (IRAK1), a down steam signaling molecule of MyD88, was decreased in the presence of PACAP. It has been reported that PACAP activates not only adenylate cyclase (AC), which eventually activates the PKA signaling pathway, but also PLC, which leads to an increase in intracellular calcium signaling. Then, we examined the effect of H89, an inhibitor of the PKA signaling pathway, and of U-73122, a known PLC inhibitor, on the increased levels of MCP-1. MCP-1 expression was reversed by H-89 but not by U-73122 as shown in Figure 5. These results indicated that PACAP suppressed the expression of MCP-1 through the cAMP/PKA-dependent signaling pathway.

### 3.4. PACAP Inhibited the Activation of ERK and NF-*κ*B Signaling

**Figure 6:**
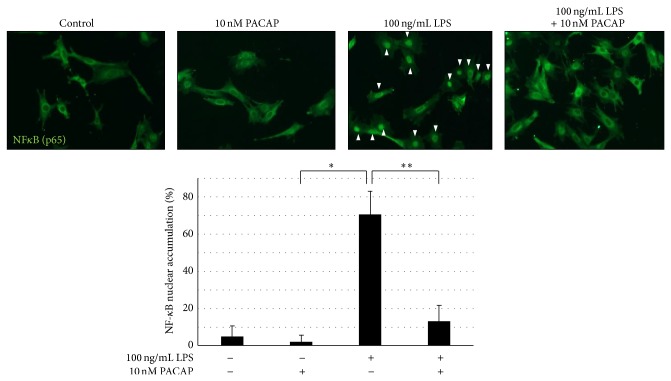
Effects of PACAP on NF-*κ*B activation in cultured podocyte. Podocytes were pretreated with 10 nM PACAP for 1 h and then stimulated with 0.1 *μ*g/mL LPS for 30 min. Cells were fixed for immunocytochemistry for evaluation of nuclear translocation of NF-*κ*B. ^*^ *P* < 0.01 versus control. ^**^ *P* < 0.05 versus LPS. Experiments were repeated at least three times and bars represented mean ± SD. Arrowheads indicate the nuclear translocation of NF-*κ*B.

**Figure 7:**
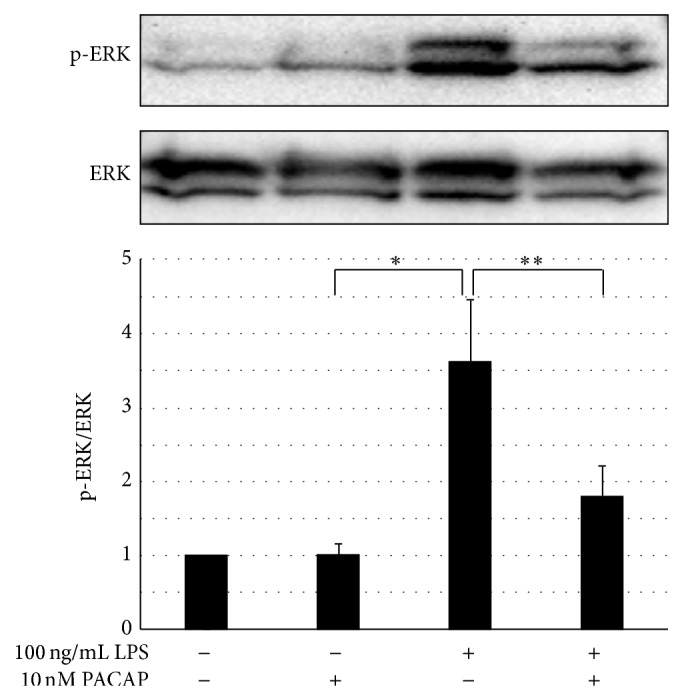
Effects of PACAP on ERK activation in cultured podocyte. Podocytes were pretreated with 10 nM PACAP for 1 h and then stimulated with 0.1 *μ*g/mL LPS for 30 min. Cells were lysed for immunoblotting and assessed for ERK1/2 phosphorylation. ^*^ *P* < 0.01 versus control. ^**^ *P* < 0.05 versus LPS. Experiments were repeated at least three times and bars represented mean ± SD.

It has been reported that LPS can activate TLR4 and subsequently the MyD88 transfers signals by activating NF-*κ*B and mitogen-activated protein kinase, which eventually results in the expression of proinflammatory cytokines. Therefore, we examined the effects of PACAP on the NF-*κ*B transnuclear localization and phosphorylation of ERK. Figures 6 and 7 showed that, in the presence of LPS, NF-*κ*B transnuclear localization and phosphorylation of ERK were significantly increased and PACAP significantly ameliorated both.

## 4. Discussion

In the present study, we reported that VPAC1, a PACAP receptor, was exclusively expressed in glomerular podocytes. PACAP, a substrate for DPP4, activated the cAMP/PKA signaling pathway in cultured podocytes and inhibited the expression of inflammatory cytokines, which were induced by LPS/TLR4 signaling.

PACAP was first identified 25 years ago and has become one of the most studied neuropeptides [11, 12]. PACAP is expressed not only throughout the nervous system but also in peripheral tissues, such as the gastrointestinal tract, the endocrine tissues, and the urinary tract. In addition to widespread expression of PACAP, it reportedly has a variety of biological functions that are primarily neuroprotective and general cytoprotective roles through the specific receptors, such as PAC1, VPAC1, and VPAC2. The renoprotective roles of PACAP have also been reported. For instance, PACAP has protected the kidney from ischemia-induced kidney injuries [14], myeloma injuries [21], cisplatin-induced renal failure [22], cyclosporine A-induced nephrotoxicity [23], and DN [18]. These renoprotective actions appeared to depend on the anti-inflammatory actions on circulating cells, glomerular cells, and tubular cells.

The kidney is injured by a wide variety of insults leading to chronic kidney diseases (CKD). Among CKD, DN is the leading cause of end-stage kidney disease. Banki et al. reported that PACAP-treated streptozotocin-induced diabetic mice had fewer histological changes, such as PAS-positive areas within the glomeruli, tubular damage, and arteriolar hyalinosis compared with the controls [18]. PACAP treatment also decreased the expression of a number of cytokines that were upregulated in diabetic conditions [18]. However, the specific cell types that are affected by PACAP directly have not been reported. Therefore, we localized the PACAP receptors in kidney. Jean CR reported that VPAC1, but not PAC1 and VPAC2, was expressed in human glomeruli [24]. In agreement with this previous report, we found that VPAC1 was expressed within the glomerulus and localized its expression to podocytes.

Recent studies of podocyte-expressed genes have considerably enhanced our knowledge of the molecular mechanisms of glomerular filtration [25]. A number of podocyte-expressed genes, such as nephrotic syndrome type-1 (NPHS-1), nephrotic syndrome type-2 (NPHS-2); actin-related proteins such as *α*-actinin 4, inverted formin 2, and CD2-associated protein (CD2AP); and cytoplasmic signaling molecules, such as phospholipase c, epsilon-1 (PLCE1) and transient receptor potential cation channel, subfamily c, member 6 (TRPC6), were all involved in maintaining the cytoskeletal dynamics. The gene mutation of these genes leads to nephrotic syndrome or glomerulosclerosis. Therefore, among the glomerular cells, the podocytes are becoming the most highlighted cell type in the field of glomerular research of most glomerular diseases. Thus, it is intriguing that PACAP may give signals to podocytes.

Because it has been reported that PACAP has anti-inflammatory effects in a wide variety of disease models, we focused on the anti-inflammatory effects of PACAP on cultured podocytes. Among the inflammatory signals, TLR, a sensor in the innate immune system [26], has been highlighted in the development of DN [27]. It has been reported that TLR2 and TLR4 have been expressed in podocytes and activated in diabetic conditions [28]. Activated TLRs have been reported to induce proinflammatory cytokines in podocytes and a disorganized podocyte cytoskeletal structure [29] which eventually led to proteinuria. Therefore, our finding about the inhibition of podocytes' TLR-related signaling in the presence PACAP is significant in terms of treating DN.

Indeed, it has been recently reported that the treatment of PACAP effectively counteracted diabetes induced podocyte injury* in vivo* [30].

TLR is a conserved family of pattern recognition receptors, which is triggered by microbial pathogens, fatty acids, uric acids, oxidative stress, and high glucose. When TLR is activated, it recruits different adaptor molecules, such as MyD88 and IRAK-1. Activated MyD88-dependent or MyD88-independent pathway engages the activation of ERK and NF-*κ*B signaling, which results in inflammatory cytokines. Because PACAP attenuated both activation of ERK and NF-*κ*B in our case, PACAP is expected to inhibit the signals upstream of those two pathways. It has reported that administration of PACAP attenuated the expression of TLRs and its adaptor protein in kidney [31]. In agreement with these previous reports, LPS-induced inflammation related genes, such as MCP-1 and IL-6, and TLR signals related genes, such as TLR2, TLR4, and MyD88 but not IRAK-1 were suppressed by PACAP in this study. It has been reported that LPS-induced expression of TLR2 has been suppressed by ciprofloxacin through the production of prostaglandin (PG) E2 but not through PKA in monocyte/macrophages [32]. Because PGE2 was reportedly induced in the presence of PACAP [33], NS398, an inhibitor of PGE2, was tested and was not able to reverse the effects of PACAP, which attenuated the TLR4 expression (data not shown). Thus, the precise mechanisms of how PACAP inhibits the expression of TLRs and MyD88 need to be further analyzed.

DPP4i has been introduced in clinic and has become one of the most promising options to treat diabetic patients owing to their effectiveness in glucose lowering and low risk of hypoglycemia and weight gain. DPP4i stabilizes its substrates and incretins (GLP-1 and GIP), which are the main substrates for lowering blood glucose. Beyond the hypoglycemic action, it has been reported that DPP4i has numerous potential benefits in diabetic vascular complications, including DN [10]. The possible renoprotective effects of DPP4i include the reduction of oxidative stress and anti-inflammation and the improvement of endothelial dysfunctions through incretin-dependent and -independent pathways. Since it has been reported that GLP-1 receptor was not detected in glomerulus [7] and we were also able to detect neither GLP-1 receptor nor GIP receptor both in glomerulus and cultured podocyte (Supplemental Figure 2: expression of GLP-1 receptor and GIP receptor were evaluated by RT-PCR), incretin independent pathway might have roles in protecting against DN. PACAP is N-terminally truncated by DPP4, and it has been reported that DPP4-degraded PACAP loses its insulinotropic effects [34]. Because the N-terminal of PACAP is a high-affinity site for VPAC1 binding [34], DPP4-degraded PACAP may reduce the signals through VPAC1. We also confirmed that the treatment of linagliptin, DPP4i, inhibited the degradation of PACAP which secreted from cultured cells (Supplemental Figure 3: linagliptin protect PACAP from degradation). In this study, we demonstrated that PACAP had anti-inflammatory effects on podocytes, leading to an assumption that DPP4i protects kidney from injuries through the stabilization of PACAP. Nevertheless, we have not confirmed that PACAP has protective roles* in vivo*. Because the half-life of PACAP injected into mice and humans is between 2 and 10 min due to enzymatic degradation [35], truncated PACAP is required to be produced in order to get PACAP more stable* in vivo* without losing receptor activating effects. And this project is under way now.

## 5. Conclusion

In conclusion, we have shown that PACAP has anti-inflammatory effects on glomerular podocytes. Because treatment options for DN are still limited, PACAP may be a good candidate for prevention/attenuation of DN. However, more study is definitely needed to prove this possibility.

## Supplementary Material

Supplemental Table 1: Primer sequences which were used for RT-PCR or Quantitative PCR.
Supplemental Figure 1: Effect of PACAP on intracellular cAMP in cultured podocytes.
Supplemental Figure 2: Expression of GLP-1 receptor and GIP receptor both in kidney and cultured podocyte.
Supplement Figure 3: Linagliptin protect PACAP from degradation.
Supplementary Material and Methods: Gene transfection and immunoblotting.
