# Supplementary material for: Pituitary Adenylate Cyclase-Activating Polypeptide Protects Glomerular Podocytes from Inflammatory Injuries
Source: J Diabetes Res. 2015 Mar 2;2015:727152. doi: 10.1155/2015/727152 (PMC4363873; doi:10.1155/2015/727152)
Supplement: Supplementary file 1 — Supplemental Table 1: Primer sequences which were used for RT-PCR or Quantitative PCR. Supplemental Figure 1: Effect of PACAP on intracellular cAMP in cultured podocytes. Supplemental Figure 2: Expression of GLP-1 receptor and GIP receptor both in kidney and cultured podocyte. Supplement Figure 3: Linagliptin protect PACAP from degradation. Supplementary Material and Methods: Gene transfection and immunoblotting. [file 727152.f1.pdf]

Supplement

Gene	Forward	Reverse	product size
PAC1	5′-AGGCAATGAGTCGAGCATCT-3′	5′-ACCTTCCAGCTCCTCCATTT-3′	270
VPAC1	5′-CGGCCACCCGACATTGGGAAG-3′	5′-CTGCSTGTGGCGCCGTTGCTG-3′	323
PACAP	5′-GAACCCCAGCCTAACTAACT-3′	5′-AGGGAATGACATGAGTTCTG-3′	183
β-actin	5′-TCGTGCGTGACATCAAAGA-3′	5′-TGGACAGTGAGGCCAGGATG-3′	429
MCP-1	5′-CCTGCTGTTCACAGTTGCC-3′	5′-ATTGGGATCATCTTGCTGGT-3′	
IL-6	5′-GTTCTCTGGGAAATCGTGGA-3′	5′-GATTGTTTTCTGCAAGTGCATC-3′	
MyD8	5′-	5′-	

8	CCAGACCAAGTTTGCACT CA-3′	CGCAGGATACTGGGAAA GTC-3′	
IRAK 1	5′- AGGTTCCACTCCCTGTTT CC-3′	5′- CTCTTTGGAGGCCAGAC ACT-3′	
TLR2	5′- TGGCTCTTCTGGATCTTG GT-3′	5′- TTTCATGGCTGCTGTGA GTC-3′	
TLR4	5′- TTCTTCTCCTGCCTGACA CC-3′	5′- TGTCATCAGGGACTTTG CTG-3′	
GAPD H	5′- AACTTTGGCATTGTGGAA GG-3′	5′- GGATGCAGGGATGATGT TCT-3′	

Table-1

Primer sequences which were used for RT-PCR or Quantitative PCR

Legends to Supplementary Figures

Supplemental Figure 1.

Effect of PACAP on intracellular cAMP in cultured podocytes.

Cellular concentration of cAMP within podocytes was measured in the presence or absence of 10 nM PACAP from 15 min to 2 h. Intracellular cAMP concentrations were

measured by non-radioactive enzymeimmunoassay using cAMP Enzymeimmunoassay (EIA) system Kit (#RPN2251; GE Healthcare) according to manufacturer's protocol.

Supplemental Figure 2.

Expression of GLP-1 receptor and GIP receptor both in kidney and cultured podocyte. Expression of GLP-1 receptor and GIP receptor were evaluated by RT-PCR. RNA from pancreas was used as a positive controls.

Supplement Figure 3

Linagliptin protect PACAP from degradation.

PACAP was overexpressed with or without Linagliptin. Expressions of PACAP in supernatants as well as cell lysates were evaluated by immunoblotting. While PACAP protein was hardly detected without Linagliptin in supernatant, PACAP proteins appeared in the presence of PACAP. These results indicated that Linagliptin might protect PACAP from degradation.

Supplementary Material and Methods

Gene transfection and immunoblotting

PACAP cDNA was subcloned into the eukaryotic expression plasmid pcDNA3 with an aminoterminal hexa-HA sequence. Transient transfection of the plasmid into Ad293 cells was performed using using Lipofectamine LTX and PLUS Reagents (Invitrogen) according to the manufacturer's instructions. Six hours after transfection, the medium was exchanged, incubated for 48 hours with or without Linagliptin and conditioned

medium (supernatant) was collected for sodium dodecyl sulfate (SDS)-PAGE analyses. Ad293 cells were lysed in SDS sample buffer and boiled for 5 min, the proteins were then separated by 12.5% SDS-PAGE, and electrophoretically transferred to nitrocellulose membranes (Hybond-ECL, GE Healthcare) essentially as described previously. After blocking nonspecific binding of the protein, the membranes were probed with antibodies against hemagglutinin (HA; dilution 50ng/mL, Roche) at 4°C overnight, and were then incubated with anti-rat (GE Healthcare) horseradish peroxidase-linked (HRP) antibodies (dilution of 1:2500), respectively, at room temperature for 1 h. Detection was achieved with an enhanced chemiluminescence system (ECL Western blotting detection reagents, GE Healthcare).

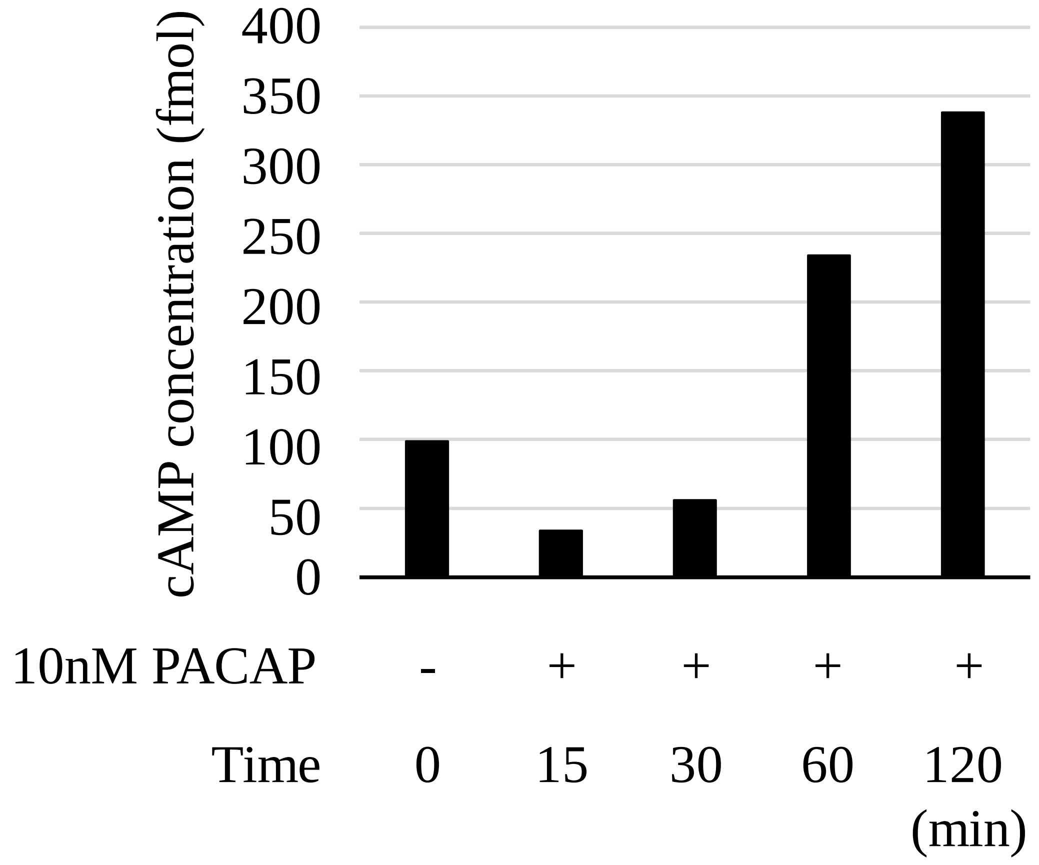

Supplementary Fig. 1

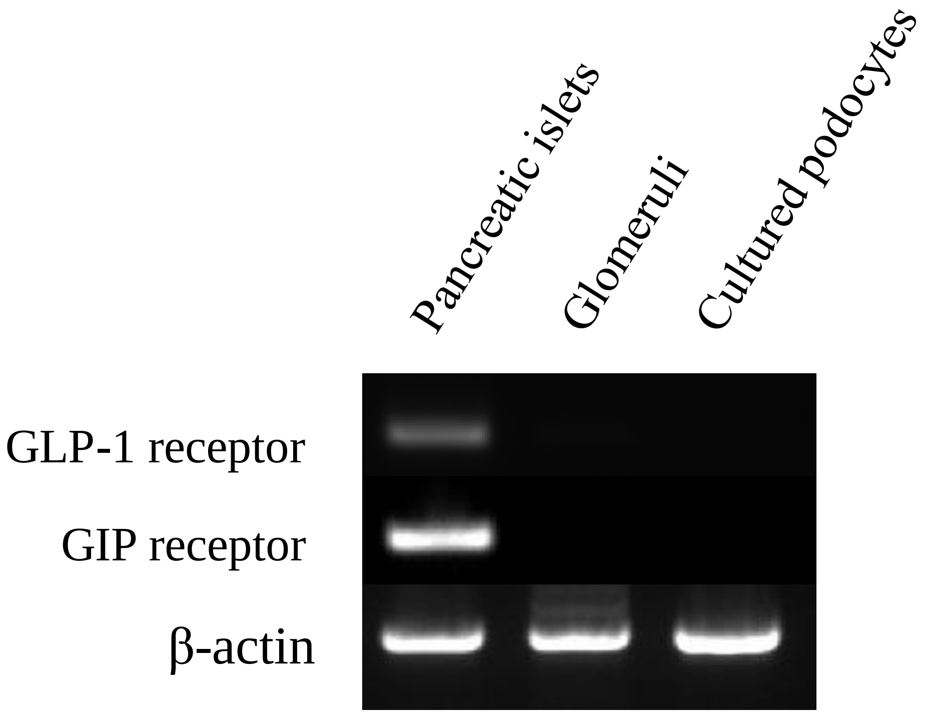

Supplementary Fig. 2

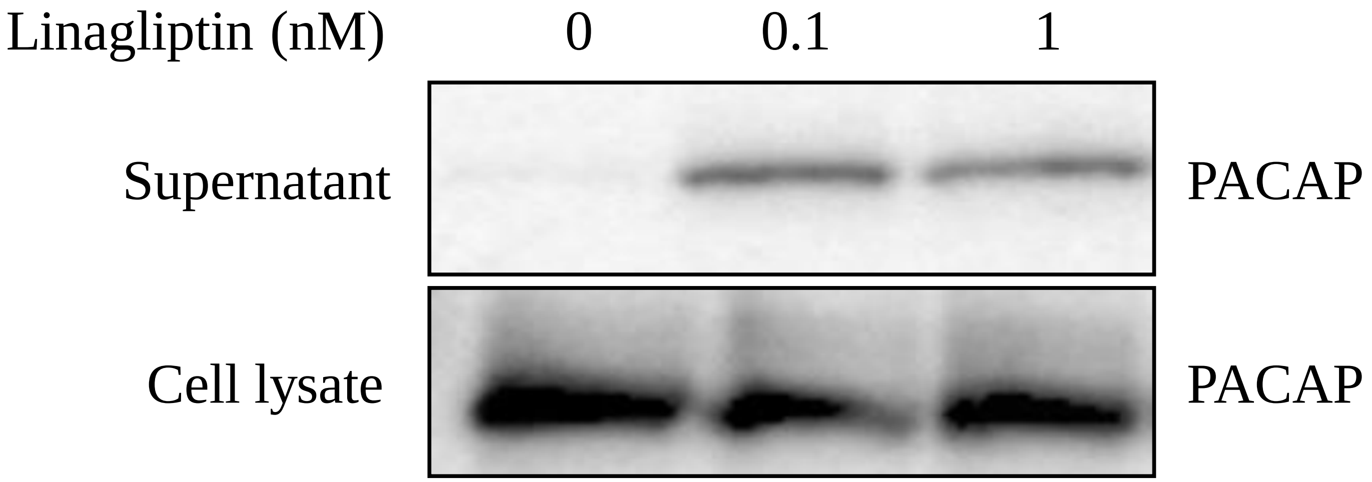

Supplementary Fig. 3
